# MIR27A rs895819 CC Genotype Severely Reduces miR-27a Plasma Expression Levels

**DOI:** 10.3390/genes15111491

**Published:** 2024-11-20

**Authors:** Georgia Ragia, Myria Pallikarou, Chrysoula Michou, Vangelis G. Manolopoulos

**Affiliations:** 1 Laboratory of Pharmacology, Medical School, Democritus University of Thrace, Dragana Campus, 68100 Alexandroupolis, Greece; mpallika@med.duth.gr (M.P.); chrymich8@mbg.duth.gr (C.M.); 2 Individualised Medicine & Pharmacological Research Solutions (IMPReS) Center, Dragana Campus, 68100 Alexandroupolis, Greece; 3 Clinical Pharmacology Unit, Academic General Hospital of Alexandroupolis, Dragana Campus, 68100 Alexandroupolis, Greece

**Keywords:** *MIR27A*, polymorphism, miR-27a-3p, expression levels, fluoropyrimidines, toxicity, response, miR-CRAFT population

## Abstract

**Background/Objectives:** *MIR27A* rs895819 polymorphism has emerged as a potential additional pharmacogenomic marker of fluoropyrimidine response. Current evidence on its potential effect on miR-27a expression, which represses DPD activity, leading to DPD deficiency and increased fluoropyrimidine-associated toxicity risk, is scarce and inconsistent. We have analyzed the effect of *MIR27A* rs895819 polymorphism on miR-27a-3p plasma expression levels under different models of inheritance to contribute further evidence on its plausible biological role in miR-27a expression. **Methods:** A total of 59 individuals with no medical history of cancer were included in this study. *MIR27A* rs895819 genotyping and miR-27a-3p expression were analyzed by using predesigned TaqMan assays. **Results**: The frequency of TT, TC, and CC genotypes was present at a prevalence of 50.8%, 44.1%, and 5.1%, respectively. Individuals carrying the CC genotype presented with decreased miR-27a-3p expression (0.422 fold-change versus TT, *p* = 0.041; 0.461 fold-change versus TC, *p* = 0.064), whereas no differences were present between TT and TC individuals (1.092 fold-change, *p* = 0.718). miR-27a-3p expression was decreased in CC individuals under a recessive model of inheritance (0.440 fold-change, *p* = 0.047). No differences were found in dominant (TT vs. TC+CC, 0.845 fold-change, *p* = 0.471) or over dominant (TT+CC vs. TC, 0.990 fold-change, *p* = 0.996) models of inheritance. **Conclusions:** *MIR27A* rs895819CC genotype leads to severely reduced miR-27a-3p expression in plasma. Further study of this association is warranted in cancer patients to apply *MIR27A* genotyping in therapeutics to identify fluoropyrimidine-treated patients who are at a decreased risk of experiencing fluoropyrimidine-induced severe toxicity.

## 1. Introduction

Dihydropyrimidine dehydrogenase (DPD) is the critical enzyme catalyzing fluoropyrimidine catabolism. The DPD enzyme is mainly expressed in the liver, which is also the main site of fluoropyrimidine metabolism. It is well known that a great interindividual variability exists in DPD activity, associated with fluoropyrimidine toxicity. The main source of interindividual variability lies within polymorphisms in the DPD-encoding gene, namely, *DPYD* [1,2]. *DPYD* is, so far, the gold-standard pharmacogene for predicting fluoropyrimidine toxicity; genotype-guided dosing guidelines exist [1], and DPYD genotyping prior of fluoropyrimidine treatment is endorsed by the European Medicines Agency [3]. Recently, in addition to the oral capecitabine (the prodrug of 5-fluorouracil), the Food and Drug Administration has approved safety labeling changes for fluorouracil injection products, suggesting physicians to consider testing for genetic variants of *DPYD* prior to initiating fluorouracil to reduce the risk of serious adverse reactions [4].

A main limitation of *DPYD* genotyping in clinical practice is the low prevalence of the deleterious polymorphisms that are currently included in genotype-based guidelines. The *DPYD* gene is under intense study for variant identification to increase the proportion of fluoropyrimidine-induced toxicity risk prediction [5,6,7]. Moreover, variants in other genes, beyond *DPYD*, are studied in an effort to build a polygenic dosing algorithm for fluoropyrimidines [8]. Additionally, other sources of DPD enzyme activity variation, including the post-transcriptional regulation of DPD expression by microRNAs, are studied as contributors of DPD-associated fluoropyrimidine-induced toxicity [9].

Several microRNAs recognize the 3’UTR of *DPYD* mRNA and may repress DPD expression [10]. miR-27a has emerged both from in silico and in vitro studies as a potential post-transcriptional regulator of DPD expression [10,11]. Overexpression of miR-27a may repress DPD activity and thus lead to DPD deficiency and increased fluoropyrimidine-associated toxicity risk [11]. Moreover, genetic variability exists in the miR-27a-encoding gene, namely, *MIR27A*; evidence exists that *MIR27A* rs895819T>C polymorphism affects miR-27a levels and is a source of variable miR-27a expression [12]. The association of *MIR27A* rs895819 variation—independently or in synergy with *DPYD* variations—with fluoropyrimidine response is being intensively studied in an effort to increase the predictive value of *DPYD* alone in fluoropyrimidine toxicity risk [2,12,13,14,15,16,17]. However, results remain inconclusive, and different genetic models of inheritance appear to have a different effect on toxicity incidence in different populations.

A main limitation for the extrapolation of *MIR27A* rs895819 to fluoropyrimidine-induced toxicity is the limited number of studies and the controversy regarding the functional role and effect of the polymorphism on miR-27a expression levels. Currently, *MIR27A* rs895819 variation has been associated either with increased or decreased miR-27a expression in different studies. Specifically, Sun et al. showed that *MIR27A* rs895819 polymorphism modulates miR-27a and its target gene *ZBTB10* levels in tumor (primary gastric carcinoma) tissue samples and presented the seminal report on C-carriage leading to increased miR-27a expression levels [18]. Offer et al. measured miR-27a expression in lymphoblastoid cell lines and showed that CC cells had approximately 50% higher miR-27a expression compared to TT homozygous, whereas intermediate expression was noted for heterozygous cell lines [11]. Lastly, contrary to previous results, Yang et al. determined the expression level of mature miR-27a in neural progenitor cells and reported significantly reduced expression levels in miR-27a-C mutants compared to WT [19]. No data are yet available on the correlation of *MIR27A* rs895819 genotypes with circulating plasma miR-27a expression levels.

Thus, to explore the potential role of *MIR27A* genotyping in fluoropyrimidine genotype guided dosing, the functional effect of *MIR27A* rs895819 polymorphism on miR-27a expression in different cell lines, malignant or healthy tissues, and other easily accessible biological fluids such as plasma, needs to be established. The aim of the present study was to analyze the effect of *MIR27A* rs895819 polymorphism on plasma expression levels of miR-27a-3p under different models of inheritance in individuals with no medical history of cancer and to add further evidence on its plausible biological role in miR expression, which can be studied in future studies on cancer patients treated with fluoropyrimidines.

## 2. Materials and Methods

### 2.1. Study Population

A total of 59 individuals with no medical history of cancer were included in this study. Due to the role of miR-27a in cancer development, a non-cancer population was chosen to study the net *MIR27A* genotype: miR-27a phenotype correlation. All individuals are participants of the miR-CRAFT study, a study designed to assess the ability of direct oral anticoagulants (DOACs) to induce epigenetic modifications, including alterations on microRNA expression in newly diagnosed atrial fibrillation patients starting DOAC therapy [20]. In the miR-CRAFT study, epigenetic modifications are followed over time and samples are drawn at 3 different timepoints: at diagnosis (baseline, t0), after 7 (t1), and 28 (t2) days of therapy with DOACs. All subjects participated after being informed about this study and giving their written consent. The study protocol was approved by Ethical Committee of Athens General Hospital “Elpis” (approval ΕΣ 23/14 April 2019) and of the Academic General Hospital of Alexandroupolis (approval ΕΣ 3/3 February 2022).

### 2.2. Nucleic Acid Purification

From all participants, approximately 6 mL blood was collected in EDTA tubes (3 mL per tube) at each visit (t0, t1 and t2). One tube was used for genomic DNA extraction (MagCore Automated Nucleic Acid Extractor, RBC Bioscience, New Tapei City, Taiwan), while the second tube was centrifuged for 10 min at 2000× *g* to collect plasma. Circulating plasma miRs were isolated by use of NucleoSpin^®^ miRNA Plasma (Macherey-Nagel, Düren, Germany). Extracted DNA and miR samples were stored at −20 and −80 °C, respectively, until use.

### 2.3. MIR27A rs895819 Genotyping

Genotyping of *MIR27A* rs895819T>C was conducted in 96-well plates on QuantStudio ™ 12K Flex Real-Time PCR System (ThermoFisher Scientific, Waltham, MA, USA) by use of TaqMan assay, as described earlier [12]. DNA purity and quantity were assessed via a UV-Vis Spectrophotometer Q5000 (Quawell, San Jose, CA, USA) and a Qubit 4 fluorometer (ThermoFisher Scientific), respectively. Approximately 20 ng of genomic DNA was used for genotyping reactions. In each 96-well plate, both non-template controls and positive controls corresponding to *MIR27A* TT, TC, and CC genotype were included. In 10% of randomly selected samples, genotyping was replicated by an independent researcher, with a 100% accordance in results. QuantStudio 12K Flex Software v1.5 was used for genotype calling. Multicomponent plots for each sample were used for the verification of amplification. In the presence of *MIR27A* rs895819T>C genotype, discrimination plots were automatically generated.

### 2.4. miR-27a-3p Expression Analysis

Based on the online database for the prediction of functional microRNA targets, miRDB [21], miR-27a-3p is the major isoform of miR-27a; thus, 3p strand was selected for the expression analysis. For miR-27a-3p expression, samples from t0 and t1 were used for the analyses described herein. miR quantity was assessed by use of Qubit 4 fluorometer (ThermoFisher Scientific) via a QUBIT MICRORNA ASSAY KIT (ThermoFisher Scientific), and all samples were diluted to a final concentration of 0.5 ng/μL. A total of 1 ng of plasma microRNAs was used to synthesize cDNA (TAQMAN ADV MICRORNA CDNA SYN, ThermoFisher Scientific). The predesigned TaqMan assay 478384_miR (ThermoFisher Scientific) was used for miR-27a-3p quantitative RT-PCR, and miR-16-5p (477860_miR, ThermoFisher Scientific), which is consistently expressed in plasma [22], was used as an endogenous control for data normalization. Reactions were carried out in 96-well plates on QuantStudio™ 12K Flex Real-Time PCR System (ThermoFisher Scientific) in 10 μL of a total reaction volume containing 5 µL of TaqMan™ Fast Advanced Master Mix, 0.5 μL of 20x TaqMan assay, 2.5 μL of 1:10 diluted cDNA template, and 2 μL RNase-free water. Conditions of qPCR were determined according to the manufacturer and consisted of an initial enzyme activation step at 95 °C for 20 s followed by 40 cycles of 95 °C for 1 s (denature) and 60 °C for 20 s (anneal/extend).

QuantStudio 12K Flex Software v1.5 was used to verify the amplification. All samples were analyzed in duplicates. Technical replicates with a Ct standard deviation greater than 1, or with an average Ct greater than 35, were omitted from further analysis. Relative quantitation (RQ) (2^−∆∆Ct^, where Ct = threshold cycle, delta Ct = Ct miR-27a-3p minus Ct miR-16-5p, Delta Delta Ct = ∆Ct interrogation group—∆Ct reference group) was calculated using ExpressionSuite Software Version 1.3 (Applied Biosystems, Carlsbad, CA, USA).

### 2.5. Statistical Analysis

The Shapiro–Wilk test was used to assess the normality of continuous variables. Continuous variables are expressed as mean ± standard deviation (SD) in the case of normal distribution; otherwise, they are expressed as a median (25th and 75th percentiles). *MIR27A* frequencies of genotypes and 95% confidence intervals (95% C.I.) were calculated using the IBM SPSS Statistics for Windows, Version 27.0 (Armonk, NY, USA: IBM Corp.). *MIR27A* allele frequency was estimated via the gene counting method. The Hardy–Weinberg equation was used to estimate departure from Hardy–Weinberg equilibrium. miR-27a-3p expression was compared via the 2^−∆∆Ct^ method to identify potential differences from t0 to t1. Since no differences were present, t2 samples were not further analyzed, and pooled t0 and t1 samples (tetraplicates) were used to compare miR-27a-3p expression within *MIR27A* genotypes. A *p*-value less than 0.05 was considered statistically significant.

## 3. Results

### 3.1. Population Characteristics

The demographic and clinical characteristics of the subject population are shown in Table 1. All individuals are newly diagnosed atrial fibrillation patients (33 male, 26 female) with mean age of 70 years (±SD 12 years). None of the subjects has a medical history of cancer since this is an exclusion criterion of the miR-CRAFT study [20]. The most common co-morbidities were hypertension (61%), type 2 diabetes (27.1%), and dyslipidemia (52.5%).

### 3.2. Frequency of MIR27A rs895819 Genotypes

In the study population, 30 individuals had the TT genotype (50.8%), 26 were CT heterozygous (44.1%), and 3 were CC homozygous (5.1%) (Table 2). *MIR27A* rs895819C allele frequency was calculated at 27.1%. The frequency of genotypes and alleles did not deviate from Hardy–Weinberg equilibrium (χ^2^ = 0.78, *p* = 0.38) and was in accordance with the prevalence previously reported in the Greek population [12].

### 3.3. miR-27a-3p Expression in Individuals After DOAC Treatment

In the cohort population, miR-27a-3p expression was not altered from baseline (t0) after 7 days of DOAC treatment (t1) (0.85 fold-change, *p* = 0.486). Since 7-days therapy had no impact on miR-27a-3p expression, t2 samples were not further analyzed, and for each individual, miR-27a-3p expression at t0 and t1 samples was pooled and used for the study of the effect of *MIR27A* rs895819 variation on miR-27a-3p expression.

### 3.4. miR-27a-3p Expression in MIR27A Genotypic Groups

**Figure 1:**
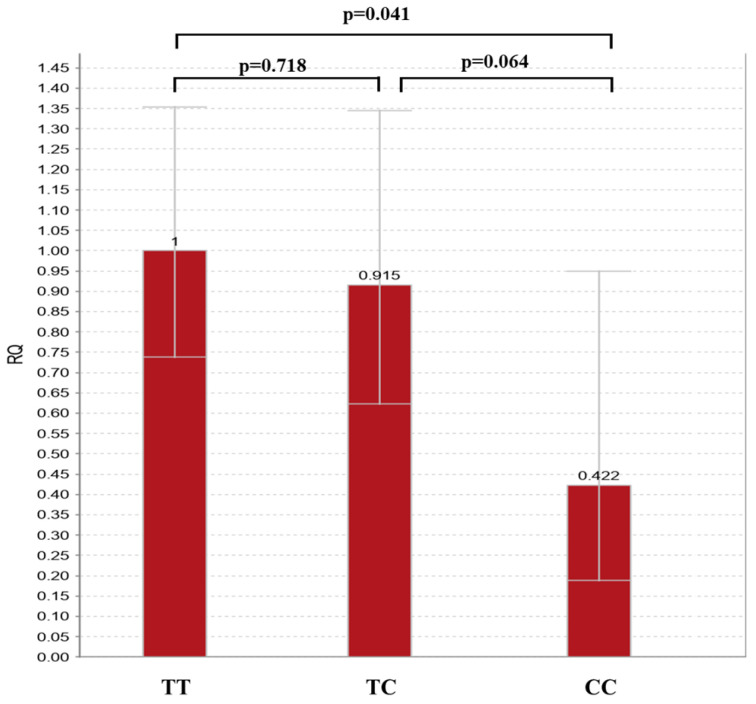
miR-27a-3p expression in *MIR27A* genotypes in pooled samples of t0 and t1 for each individual. Error bars represent the range of relative quantitation (RQ) values. RQ, relative quantitation.

Comparison of miR-27a-3p expression levels within *MIR27A* genotypes has shown that individuals carrying the CC genotype presented with nearly 60% decreased expression of miR-27a-3p versus TT (0.422 fold-change, *p* = 0.041) or TC (0.461 fold-change, *p* = 0.064) individuals (Figure 1). TT and TC individuals had similar miR-27a-3p expression levels (1.092 fold-change, *p* = 0.718) (Figure 1).

**Figure 2:**
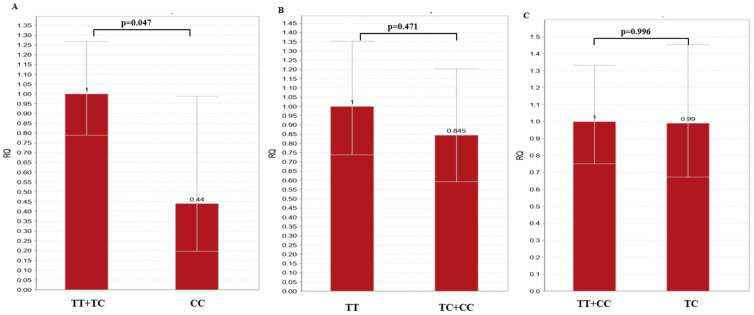
miR-27a-3p expression in *MIR27A* models of inheritance: (**A**) recessive model; (**B**) dominant model; (**C**) over dominant model. Error bars represent the range of relative quantitation (RQ) values. RQ; relative quantitation.

In the recessive model of inheritance (TT+TC vs. CC), miR-27a-3p expression was decreased in CC individuals (0.440 fold-change, *p* = 0.047) (Figure 2A). No differences were present in the *MIR27A* dominant (TT vs. TC+CC, 0.845 fold-change, *p* = 0.471) or over dominant (TT+CC vs. TC, 0.990 fold-change, *p* = 0.996) model of inheritance (Figure 2B and Figure 2C, respectively).

## 4. Discussion

In the present study, we have assessed the effect of *MIR27A* rs895819 polymorphism on miR-27a-3p plasma expression levels. We have found that *MIR27A* homozygous CC genotype severely impacts miR-27a-3p expression, leading to a nearly 60% reduction compared to TT or TC individuals. To the best of our knowledge, this is the seminal study that reports such an effect on miR-27a plasma expression levels.

miR-27a, encoded by *MIR27A* gene, has been intensively studied in tumor development and metastasis [23]. It exerts carcinogenic effects in several cancers; thus, it has emerged as a microRNA of potential prognostic and diagnostic importance in cancer diagnosis [24,25,26]. In addition to cancer development, miR-27a is acknowledged as a contributing factor to toxicity incidence to fluoropyrimidines via inhibition of *DPYD* expression [10,27]. Increased miR-27a expression has been associated with severely reduced DPD activity and subsequent fluoropyrimidine-induced severe toxicity, whereas reduced miR-27a expression in cancer cells can potentially lead to tumor resistance to therapy [10].

Evidence exists that a *MIR27A* polymorphism, namely, rs895819T>C, is an additional source of DPD activity variability through its functional effect on miR-27a expression [28]. This polymorphism has been assessed in several studies for its association—independently or in synergy with *DPYD* variations—with toxicity incidence to fluoropyrimidines; however, to date, the results on its effect on fluoropyrimidine therapy and on its use as a pharmacogenomic marker of response are still inconsistent. More specifically, results so far show that *MIR27A* rs895819T>C polymorphism is associated, independently, with chemotherapy-induced adverse events under recessive (CC genotype) [14], dominant (TC+CC genotypes) [15], or over dominant (TC genotype) [12,13] models of inheritance, or that *MIR27A* rs895819T>C association with FP toxicity is dependent on *DPYD* risk variant allele status [15,16,17]. In a recent meta-analysis, *MIR27A* rs895819 was not associated with 12-week hematological or digestive > grade 4 toxicity, irrespective of *DPYD* variants [2]. Whereas several differences in study design and in the toxicity classification may explain the discrepancy in the results, a main source of the obscure association of the *MIR27A* rs895819 variant with fluoropyrimidine response is the lack of solid evidence on its effect on miR-27a expression.

Genetic variants in microRNA-encoding genes may have an impact on the transcriptional profile of target genes [29,30]. The mechanism underlying the *MIR27A* rs895819T>C effect on miR-27a expression is largely unknown. The polymorphism is located in the terminal loop of miR-27a, resulting in a two base-larger hairpin [31], suggesting that it may influence the expression levels of mature miR-27a without impairing its binding affinity with target mRNAs [29]. This structural modification has been linked to two opposite effects on miR-27a expression. It has been proposed that larger hairpin loop regions can be more effectively processed, leading to more effective miR-27a maturation [11]; and on the other hand, it has also been proposed that the C allele could affect the maturation process and stability of miR-27a, thus leading to reduced expression of mature and precursor miR-27a [19]. The potential association of *MIR27A* rs895819T>C variant with miR-27a expression levels has been assessed only in three studies, each showing a different effect of the *MIR27A* genotypes on miR-27a expression. Sun et al. analyzed the expression of miR-27a in 65 tumor tissue samples of primary gastric sarcoma of Han Chinese tissue donors [18]. The prevalence of *MIR27A* rs895819 CC cases was 15.4%. The authors have found that the relative Ct ratio (miR-27a Ct/U6 Ct) was lower in CC samples, suggesting that lower Ct represents a higher expression [18]. Offer et al. measured miR-27a expression in lymphoblastoid cell lines previously genotyped for rs895819 [11]. CC homozygous cells had approximately 50% higher miR-27a expression, and this result was replicated in HEK293T/c17 cells expressing constructs for the rs895819C allele [11]. Yang et al. determined the expression levels of pre-miR-27a and of mature miR-27a in neural progenitor cells [19]. Both in mature and progenitor miR-27a, the C allele led to significantly reduced expression levels [19]. While these studies have analyzed miR-27a expression in tissues and cell lines, the effect of *MIR27A* polymorphism on miR-27a plasma levels has not been assessed to date. In our study, we have correlated the *MIR27A* rs895819 variation with miR-27a-3p circulating plasma levels, and we have found that CC genotype severely impacts miR-27a-3p expression, leading to less than 50% expression compared to TC and TT genotypes. This finding is in line with the results published by Yang et al. [19]. Interestingly, both studies have assessed miR-27a-3p expression in non-malignant material.

It has been extensively described that in cancer, microRNA biogenesis pathways are deregulated, resulting in the up- or down-regulation of microRNAs in cancer cells [32]. Specifically, for miR-27a, different expression features have been shown in tumor cells for various cancer types [33]. Thus, it cannot be ruled out that the effect of *MIR27A* rs895819 polymorphism on miR-27a expression is differentially reflected between healthy and cancerous samples. A potential explanation for this discrepancy is the fact that in cancer, additive stimuli, including hypoxia, inflammation, and the tumor microenvironment, may increase miR-27a expression [32]. Whether tumor miR-27a content is correlated with plasma circulating expression levels needs to be further examined. Since efforts are focusing on using microRNAs as predictive and/or prognostic biomarkers for cancer patient prognosis, several advances and linkage of genomics to miRNomics are awaited in the field.

In the present manuscript, the correlation of the *MIR27A* CC genotype to severely reduced miR-27a plasma expression we have identified provides evidence for the biological role of the polymorphism in miR-27a-3p plasma expression in individuals with no medical history of cancer; however, it cannot be determined whether this association reflects the liver content of miR-27a or if it linearly affects DPD activity and thus the on-site fluoropyrimidine catabolism and toxicity incidence. Nonetheless, we have previously shown in a cancer patient cohort consisting of 313 fluoropyrimidine-treated patients that *DPYD* polymorphisms predict any-grade and severe toxicity incidence with high specificity (100% and 98%, respectively); however, their low frequency results in a low sensitivity of analysis (4% and 12%, respectively) [34]. In the same population, *MIR27A* rs895819 polymorphism was associated—independently of *DPYD* variants—with fluoropyrimidine-induced grade 3–4 toxicity under the over dominant model of inheritance, and we have shown that none of the CC individuals experienced severe toxicity (percentage of CC in patient cohort was 11.5%; 25 patients developed grade 3–4 toxicity in the total population) [12]. Extrapolation of the current findings on the reduced expression of miR-27a-3p in CC individuals is in line with the absence of severe toxicity in CC fluoropyrimidine-treated cancer patients. However, we do not confirm that miR-27a-3p expression in TC individuals surpasses the miR-27a-3p expression in TT individuals.

Our study has several strengths. The study population consists of participants of the miR-CRAFT study, a rather homogeneous atrial fibrillation patient population; multiple sampling was available for each participant according to the study design [20]. miR-27a-3p expression analysis, at two different timepoints, showed that its expression was stable at a time interval of 7 days, in which patients were treated with DOACs. Therefore, the reduced expression of miR-27a-3p seen in CC individuals can be attributed, per se, to *MIR27A* variant. Additionally, we have shown that the genotype-attributed altered miR-27a-3p expression can be identified in plasma, an easily accessible biological fluid, thus linking a genetic to an epigenetic biomarker. However, we should also acknowledge that the population studied is cancer-free; thus, no tumor samples were available to correlate the expression of miR-27a-3p in both plasma and tissues. Additionally, the *MIR27A* rs895819CC genotype is rather scarce in frequency (5.1% in the present study). The results of the present study require validation in larger cohorts of both cancer and non-cancer individuals to shed further light on the mechanism underlying the effect of *MIR27A* variation on miR-27a expression and on the correlation of plasma and tumor miR-27a expression.

## 5. Conclusions

In conclusion, our results show that the *MIR27A* rs895819CC genotype leads to severely reduced miR-27a-3p expression in the plasma of individuals with no medical history of cancer. Our results need to be validated in larger studies including fluoropyrimidine-treated cancer patients of different ethnicities. If the conclusion that *MIR27A* rs895819 genotyping reflects the content of miR-27a is replicated, then identifying *MIR27A* CC individuals can be extended in therapeutics to identify fluoropyrimidine-treated patients who are at decreased risk of experiencing fluoropyrimidine-induced severe toxicity. Further studies correlating miR-27a-3p plasma and cancer tissue expression will help in improving drug safety and patient response.

## Figures and Tables

**Table 1:** Demographic and clinical characteristics of patient population (*n* = 59).

Demographic and Clinical Characteristics	Values
Male (*n*, %)	33 (55.9)
Age (years, mean ± SD)	70 ± 12
Weight (kg, median, 25th, 75th percentiles)	78 (62, 87)
Height (cm, median, 25th, 75th percentiles)	168 (155, 175)
Smokers (*n*, %)	12 (20.3)
Hypertension (*n*, %)	36 (61.0)
Type 2 Diabetes (*n*, %)	16 (27.1)
Dyslipidemia (*n*, %)	31 (52.5)

SD; standard deviation.

**Table 2:** Prevalence of *MIR27A* genotypes in total cohort.

*MIR27A* rs895819	Total Cohort (*n* = 59)	
	n (%)	95% C.I.
**Genotypes**		
TT	30 (50.8)	38.3–63.3
TC	26 (44.1)	31.9–56.8
CC	3 (5.1)	1.5–12.9
**Recessive model**		
TT+TC	56 (94.9)	87.1–98.5
CC	3 (5.1)	1.5–12.9
**Dominant model**		
TT	30 (50.8)	38.3–63.3
TC+CC	29 (49.2)	36.7–61.7
**Over dominant model**		
CC+TT	33 (55.9)	43.2–68.1
TC	26 (44.1)	31.9–56.8

## Data Availability

The original contributions presented in this study are included in the article. Further inquiries can be directed to the corresponding authors.
